# Effectiveness of digital screening tools in detecting cognitive impairment among community-dwelling elderly in Northern China: A large cohort study

**DOI:** 10.1016/j.tjpad.2025.100080

**Published:** 2025-02-07

**Authors:** Xiaonan Zhang, Feifei Zhang, Sijia Hou, Chenxi Hao, Xiangmin Fan, Yarong Zhao, Wenjing Bao, Junpin An, Shuning Du, Guowen Min, Qiuyan Wang, Wencheng Zhu, Yang Li, Hui Zhang

**Affiliations:** a Department of Neurology, First Hospital of Shanxi Medical University, Taiyuan, 030001, China; b Department of Radiology, First Hospital of Shanxi Medical University, Taiyuan, 030001, China; c Department of Medical Imaging, Shanxi Medical University, Taiyuan, 030001, China; d Shanxi Key Laboratory of Intelligent Imaging and Nanomedicine, First Hospital of Shanxi Medical University, Taiyuan, 030001, China; e Department of the First Clinical Medicine, Shanxi Medical University, Taiyuan, 030001, China; f Institute of Software Chinese Academy of Sciences, Beijing, 100101, China; g CAS-Ruiyi Information Technology Co., Ltd, Beijing, 100089, China

**Keywords:** Cognitive impairment, Digital screening tool, Behavioral tool, Machine learning model, Plasma biomarker

## Abstract

**Introduction:**

This study assessed the effectiveness of three digital screening tools in detecting cognitive impairment (CI) in a large cohort of community-dwelling elderly individuals and investigated the relationship between key digital features and plasma p-tau217 levels.

**Methods:**

This community-based cohort study included 1,083 participants aged 65 years or older, with 337 diagnosed with CI and 746 classified as normal controls (NC). We utilized two screening approaches: traditional methods (AD8, MMSE scale, and APOE genotyping) and digital tools (drawing, gait, and eye tracking). LightGBM-based machine learning models were developed for each digital screening tool and their combination, and their performance was evaluated. The correlation between key digital features and plasma p-tau217 levels was analyzed as well.

**Results:**

A total of 21 drawing, 71 gait, and 35 eye-tracking parameters showed significant differences between the two groups (all p < 0.05). The area under the curve (AUC) values for the drawing, gait, and eye-tracking models in distinguishing CI from NC were 0.860, 0.848, and 0.895, respectively. The combination of eye-tracking and drawing achieved the highest classification effectiveness, with an AUC of 0.958, and accuracy, sensitivity, and specificity all exceeded 85%. The fusion model achieved an AUC of 0.928 in distinguishing mild cognitive impairment (MCI) from NC. Additionally, several digital features (including two drawing, ten gait, and one eye-tracking parameters) were significantly correlated with plasma p-tau217 levels (all |r| > 0.3, p < 0.001).

**Discussion:**

Digital screening tools offer objective, accurate, and efficient alternatives for detecting CI in community settings, with the fusion of drawing and eye-tracking providing the best performance (AUC = 0.958).

## 1 Introduction

The global dementia population is projected to increase to 152 million by 2050, primarily due to global aging trends [1]. This increase poses a significant mental and economic burden on caregivers, the healthcare system, and society [2]. Mild cognitive impairment (MCI) is considered an intermediate stage between normal aging and the onset of dementia [3]. In China alone, it is estimated that there are approximately 15.07 million dementia patients and 38.77 million MCI patients aged over 60 years [4], making it the country with the largest population of individuals with CI. However, the active consultation rate is only 12.9%, with even lower rates observed in rural areas [5]. The low awareness of the disease, high cost of advanced diagnostic techniques (*e.g.*, MRI and FDG-PET), and the low acceptance of invasive procedures (*e.g.*, lumbar puncture) in community settings create significant barriers to the early detection of CI [4]. Traditional paper-and-pencil tests, such as the Ascertain Dementia 8-item questionnaire (AD8) [6] and the Mini-Mental State Examination (MMSE) [7,8], are widely used and well-documented screening tools [9]. However, these tests are time-consuming and require administration by trained personnel [10]. Additionally, most language-based screening tools are influenced by factors such as age and education level [[10], [11], [12]]. Therefore, there is an urgent need to identify non-invasive, low-cost, and reliable markers to overcome the limitations of existing methods in community screening.

In recent years, digital technologies at the intersection of medicine and artificial intelligence have been increasingly applied to cognitive assessments. These include digitally improved scales, such as digital drawing tests [[13], [14], [15], [16]], and behavioral tools that monitor walking posture and eye movement trajectories [[17], [18], [19]]. Digital techniques can systematically quantify numerous performance details to enhance assessments, offering a more detailed perspective on task completion. Previous studies have shown that patients with amnestic mild cognitive impairment (MCI) exhibit various deficits in clock drawing tasks, such as prolonged pause time, increased stroke number, slower speed, reduced area, and incorrect clock hands, which can effectively differentiate CI from normal individuals [14,20]. Additionally, the clock composite score was correlated with atrophy of the gray matter and hippocampus [21]. A recent study demonstrated that digital clock scores were associated with greater amyloid and tau deposition in the brains of normally aging elderly individuals and provided better differentiation between Aβ+ and Aβ− groups [22]. Therefore, it is an effective tool for detecting early CI in AD trajectory.

Gait posture is also associated with CI. This association may be partially explained by shared cortical networks, such as prefrontal and temporal regions activated, which are activated during physical exercise [23,24]. Impaired spatial navigation and visuospatial function in patients with Alzheimer's disease (AD) may also impair their postural control while walking [25,26]. Several studies have shown that patients with AD and MCI exhibit various gait abnormalities, including high variability, slow walking speed, decreased stride length, and pace asymmetry [[27], [28], [29], [30]]. Pace asymmetry was negatively correlated with the Montreal Cognitive Assessment (MoCA) score [27]. Turn time in dual-task cost (DTC) was significantly correlated with plasma p-tau181 levels [31]. Therefore, gait analysis can serve as an initial tool to identify individuals at high risk for cognitive decline.

Eye tracking technolog also has the potential to detect subtle cognitive decline [[32], [33], [34]]. Eye movements are initiated and regulated by a complex neural network involving numerous cortical and subcortical regions [35,36], which undergo specific pathological changes decades before the clinical symptoms of AD appear [37]. Patients with CI exhibit deficits in attention and inhibitory control, which can be detected through eye movements during visual tasks [38]. These tasks typically involve smooth pursuit, fixation, and saccades [39,40]. Previous studies have shown that the latency in horizontal and vertical anti-saccade tasks can effectively distinguish patients with MCI and dementia from normal individuals [41]. Compared with healthy controls, patients with AD exhibit shorter fixation times, slower tracking speeds, and higher error rates [40,42].

However, in the context of a large community cohort, the discriminant validity of drawing, gait, and eye tracking tools in detecting CI remains unclear, particularly whether the integration of drawing and eye tracking assessments can improve the ability to identify patients with MCI. Previous studies have been limited by small sample sizes and the use of a narrow range of digital tools, which have affected the reliability and generality of their results. A small clinical study demonstrated that multimodal data combining speech, gait, and drawing effectively classified AD, MCI, and normal individuals [43]. Additionally, a recent community cohort study validated the effectiveness of a fusion model based on dual-task, gait, and eye tracking assessments for detecting CI, reporting an AUC of 0.987 [31]. However, the AUC for distinguishing patients with MCI from normal individuals dropped to 0.742. Furthermore, the association between key digital features and plasma p-tau217 levels poorly understood.

This study involved a large cohort of 1083 participants, from whom we collected various indicators, including drawing, gait, and eye-tracking parameters, cognitive scores, APOE genotyping, and plasma p-tau217 data. We developed three digital screening tools and their fused machine learning classification models to evaluate their potential for distinguishing CI/MCI from normal individuals. Given that plasma p-tau217 is an established diagnostic biomarker for AD [44] and reliably predicts brain amyloid levels [45], we also examined the correlation between key digital features and plasma p-tau217 levels. We hypothesize that these digital screening tools will exhibit superior discriminative performance compared to traditional methods in detecting CI and MCI.

## 2 Methods

### 2.1 Study population

**Fig. 1:**
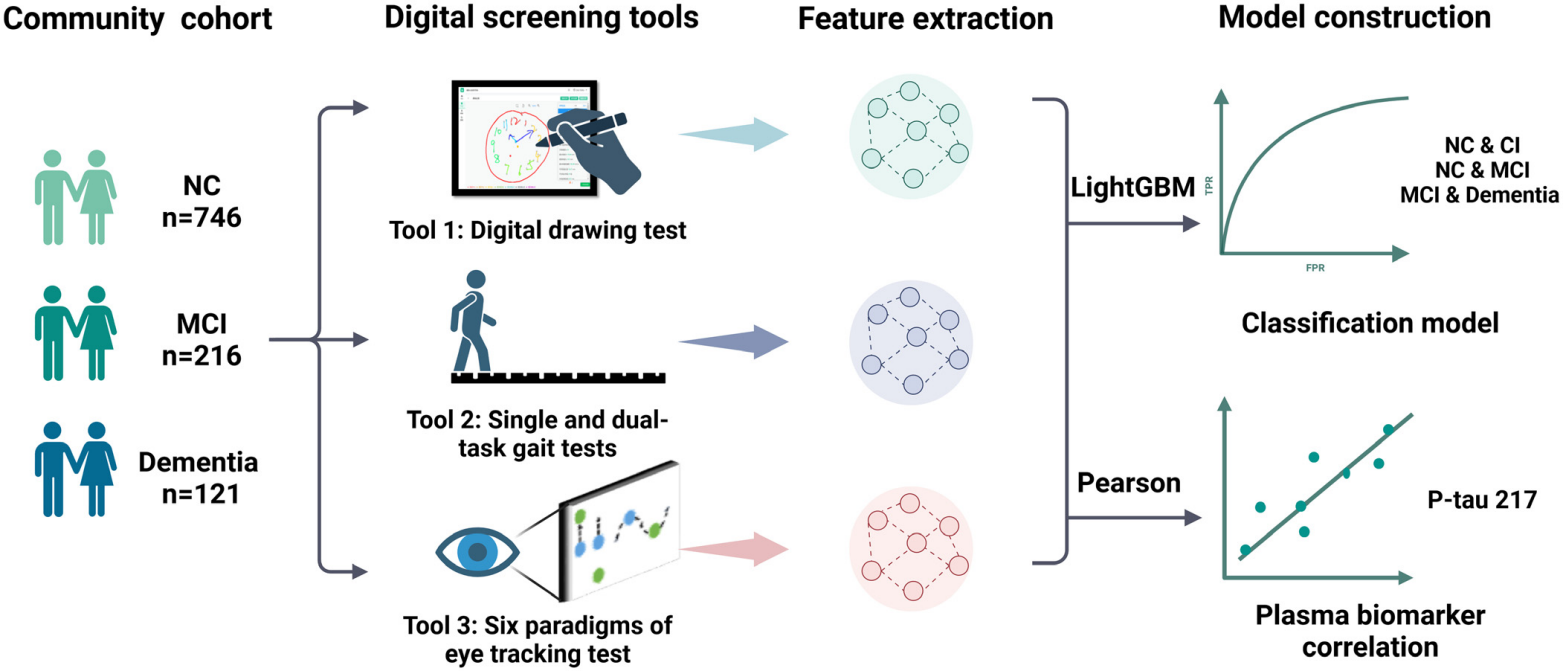
Workflow for validating the effectiveness of three digital screening tools in community cohort studies. NC, normal control; CI, cognitive impairment; MCI, mild cognitive impairment; p-tau217, phosphorylated tau 217; LightGBM, light gradient boosting machine.

**Fig. 2:**
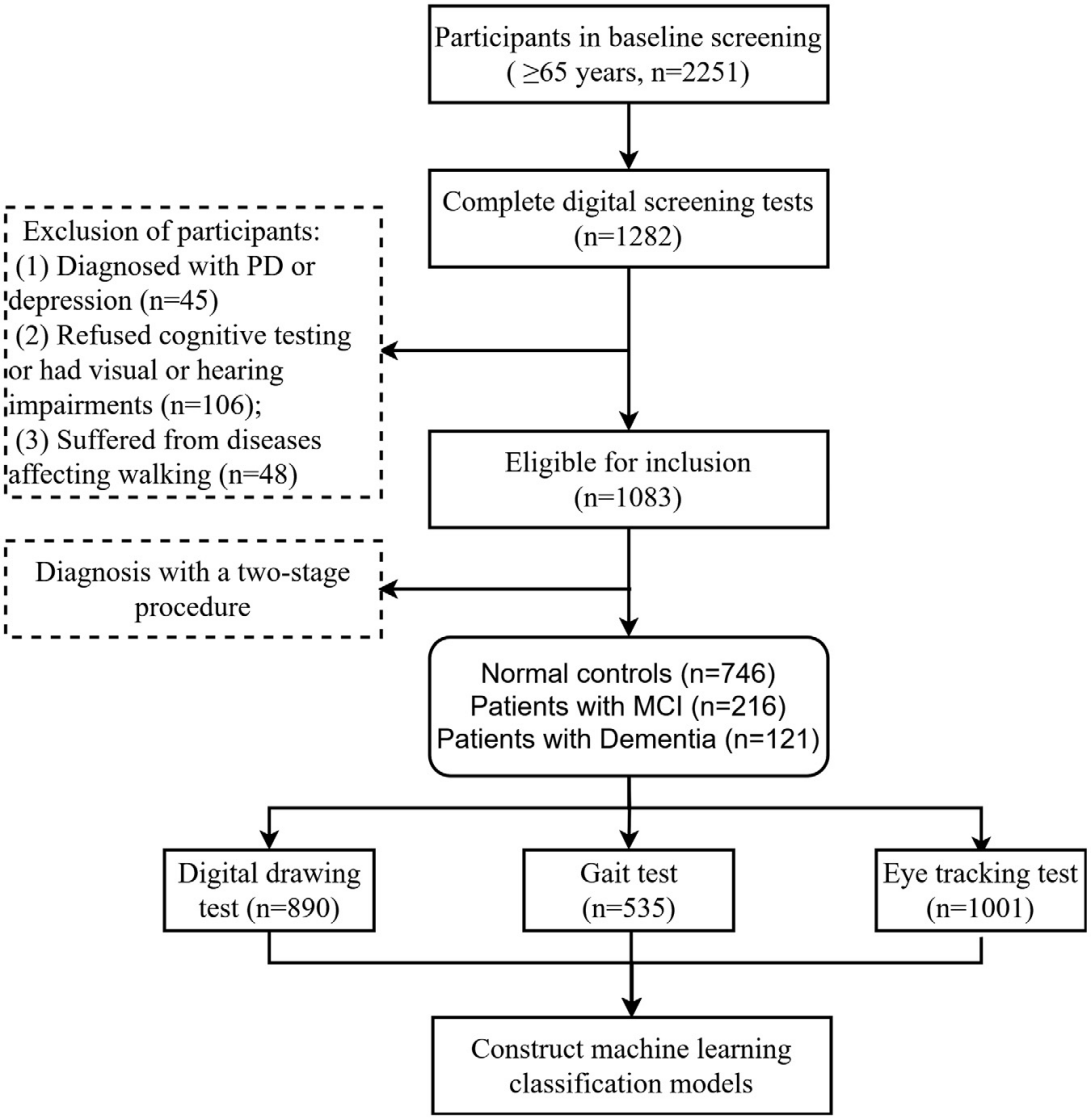
Flowchart of participant recruitment. PD, Parkinson's disease; MMSE, Mini-Mental State Examination; ADL, Activity of Daily Living scale.

This study follows the Standards for Reporting of Diagnostic Accuracy (STARD) guidelines [46]. This cross-sectional study is part of an ongoing, community-based prospective cohort study in Shanxi Province, China. Between June and October 2023, cluster sampling was used to select six communities (one urban and five rural) in Shanxi Province. A total of 2,251 residents aged 65 and older participated in the baseline screening. Enrollment coverage, based on government data for the elderly population, reached 82.5%. Of these, 43% were assessed using an in-home questionnaire alone, while 1,282 participants completed assessments using digital tools at the same time (Fig. 1). The inclusion criteria were: (1) Age≥ 65 years at the time of enrollment; (2) Han Chinese who were registered in the community system and had resided continuously in the target community for at least one year before to the survey; (3) Participants or their legal guardians provided written informed consent. The exclusion criteria were: (1) Participants who refused neuropsychological testing or were unable to complete the assessments due to visual or hearing impairments; (2) Participants with a history of traumatic brain injury, cataracts, stroke, or severe osteoarthritis that substantially impaired mobility; (3) Individuals with depression, identified based on a GDS-15 score≥10 [47], were excluded; (4) The study also included an ongoing Parkinson's disease (PD) screening as part of the cohort. Initially, individuals at risk for PD were identified using the PD screening scale [48] (score≥3), followed by further diagnostic evaluation conducted by two experienced movement disorder specialists. Ultimately, 1083 participants were eligible for inclusion. Of these, 890 completed the drawing scale, 535 the gait test, and 1001 the eye tracking test. A flowchart of participant enrollment is shown in Fig. 2.

This study protocol was approved by the Ethics Committee of the First Hospital of Shanxi Medical University (NO. KYLL-2023–123), and written informed consent (electronic version) was obtained from all participants or their guardians. The study adhered to the ethical guidelines outlined in the Declaration of Helsinki.

### 2.2 Data collection and CI diagnosis

We collected data on participants' demographic characteristics, cognitive test results, laboratory findings, medical history, and scores on the Hachinski Ischemia Scale (HIS) [49] and the 15-item Geriatric Depression Scale (GDS-15). Peripheral blood samples were collected after an overnight fast. The diagnosis of CI followed a two-stage process, which included the AD8, Mini-Cog, MMSE, and Clinical Dementia Rating (CDR) scales [50]. Assessments were conducted by trained investigators, including neurologists and nurses from various communities and provincial hospitals, all of whom underwent standardized training in neuropsychological assessments before screening. These assessments were performed using an electronic system. In the first stage, community interviewers conducted preliminary screening using the AD8 and Mini-Cog scales. Individuals with an AD8 score≥2 [6] or a Mini-Cog score<3 [51] were classified as being at risk for CI, whereas those whose scores were within the normal range on both scales were identified as normal controls(NC). In the second stage, individuals at risk for CI underwent further evaluation using the MMSE, ADL, and CDR scales to determine overall cognitive status and provide a definitive diagnosis (NC, MCI, or dementia).

The CI group included participants diagnosed with MCI or dementia, characterized by a CDR score ≥0.5. Based on Peterson's criteria [52] and the Diagnostic and Statistical Manual of Mental Disorders, Fourth Edition (DSM-IV) [53], MCI is diagnosed when: (1) cognitive impairment is reported by the subject or an informant, or identified by a trained clinician; (2) objective evidence shows impairment in one or more cognitive domains; (3) instrumental ADL may be slightly impaired, but basic ADL remains independent; (4) there is no diagnosis of dementia (CDR=0.5). For dementia diagnosis, the CDR score is>0.5, and the ADL score is significantly higher. The cognitive decline cannot be attributed to delirium or any other mental illness. Participants without signs of MCI or dementia were placed in the NC group.

### 2.3 Digital drawing test

The digital drawing test was developed by our team in collaboration with Shanxi Infreisi Technology Co., Ltd., and administered on tablet devices using a digital pen (Fig. S1A), as described in previous research [54]. The test is based on the well-known Mini-Cog scale, which includes a clock drawing subtest and a word recall subtest. The procedures and instructions are similar to the traditional paper-and-pencil version, with only the clock drawing subtest optimized for digital use. The digital version enables the extraction of multiple target parameters from the drawing process and reproduces the drawing trajectory (see supplementary materials). The test consists of three steps: (1) the evaluator clicks the audio button to randomly play one of the six word lists and instructs the subject to memorize the words. (2) the subject uses a stylus to draw a clock indicating "11:10″ on the tablet; (3) the subject recalls the three words memorized in Step 1. The input variables for constructing the drawing model include word recall scores and various drawing parameters. Correct recall of 0, 1, 2, or 3 words corresponds to a score of 0 to 3 points. The device can collect 27 feature parameters, including stroke length, pause time, drawing time, speed, number, clock face area, among others. (See Table S2 for parameter definitions). It takes about 1∼2 min.

### 2.4 Gait assessment

Our study utilized a quantitative motor function evaluation system (ReadyGo^TM^, Beijing CAS-Rui Yi Information Technology Co., Ltd.) to collect participants' gait parameters (Fig. S1B). This compact all-in-one device occupies less than 1 m² of floor space, meaning no specific room is required for testing. A set of cameras at the front of the device uses depth vision technology to capture the positions of human bone landmarks and identify critical motion nodes in real time, eliminating the need for wearable sensors. Participants were instructed to walk naturally along a 3-m straight path within a 1 × 5 m effective area in front of the camera, completing 3 round trips. Three gait tests were conducted for each participant. Initially, participants' baseline gait parameters were measured without a cognitive load. For dual-task tests, participants were asked to complete two additional cognitive tasks while walking: counting down from 100 and animal naming. The order of the three gait tests was predetermined. Only one trial per task was allowed to minimize the learning effect and ensure balance. Additionally, we assessed the impact of the cognitive task challenge on gait performance using the dual-task cost (DTC), calculated as *[(single task- dual task)/single task] × 100%*. It takes about 3∼5 min.

### 2.5 Eye tracking assessment

An intelligent virtual reality(VR) eye-tracking analysis and evaluation system (EyeKnow^TM^, Beijing CAS-Rui Yi Information Technology Co., Ltd.) was used to capture eye movement trajectories. The system comprises a control terminal (tablet) and a capture terminal (VR headset, Fig. S1C). The test requires only a chair and a table for completion. Prior to the test, participants' eye positions were calibrated using a nine-point calibration procedure to ensure central positioning within the acquisition area. For participants with aging or myopic eyes, the test would continue if they could complete the calibration while wearing glasses. Otherwise, the test stops. At the beginning of the test, a green dot would appear in the center of the VR headset display, and the subject only needs to make corresponding eye movements according to the instructions. Eye movement tasks encompass smooth pursuit (SP), median fixation (MF), lateral fixation (LF), gap pro-saccade (GPS), overlap pro-saccade (OPS), and anti-saccade (AS). It takes about 5–7 min. (1) In the SP task, a green dot moves horizontally along a sinusoidal path, with an amplitude of 20° and a speed of 10°/s, requiring participants to maintain a continuous gaze. Parameters such as pursuit speed, acceleration, offset number, and total offset could be analyzed. (2) In fixation tasks, which include MF and LF, participants were required to fixate on a stationary green target point until it disappeared. The MF lasts for 10 s, while the LF extends for 30 s at a deviation angle of 15°. Parameters such as the offset number, total offset, and offset time are analyzed. (3) The Pro-saccade tasks (including GPS and OPS). In the GPS task, a center point appears and disappears after 0.8 s, followed by a 0.2-s gap before a target point appears randomly at a 20° deviation in the top, bottom, left, and right positions. OPS differs in that the center point lasts for 1.2 s with the target point appearing 0.2 s before its disappearance, creating an 0.2-s overlap. Participants need to move their eyes quickly and accurately to the target point. The critical parameters examined in the PS tasks included saccade accuracy, latency, total time, and speed. (3) The AS task initiates with a center point that disappears after 1 s, followed by a target point appearing randomly at a 20° deviation. The participant should execute a saccade in the opposite direction to the appearing target. The AS task, in contrast to PS tasks, increases the error correction rate and error correction time analysis. (See Table S3 for parameter definitions)

### 2.6 Plasma p-tau217 detection and APOE genotyping

Venous blood was collected into tubes containing ethylenediaminetetraacetic acid (EDTA) and then centrifuged at 4 °C × 2000g for 10 min. The plasma was aliquoted into polypropylene tubes in 500 μL portions and stored at -80 °C. The average time from blood collection to freezing was <2h. Prior to analysis, the samples were subjected to only one freeze-thaw cycle. Plasma p-tau217 levels were measured using ultrasensitive Simoa technology (Quanterix, MA, US) on the automated Simoa HD-X platform (GBIO, Tianjin, China), following the manufacturer's protocol (Cat No: 104,371). The instrument diluted 100 μL of plasma at a 1:4 ratio for detection purposes. All samples were analyzed using kits from the same batch, and the operator was blinded to the participants' disease diagnoses. A total of 100 participants (50 NC and 50 MCI) underwent the plasma p-tau217 assay. Apolipoprotein E (APOE) genotyping was performed using EDTA-anticoagulated whole blood samples, without any freeze-thaw cycles. Samples (10 μL) were diluted and processed using the SNP-U24 kit (Xi'an Tianlong Technology Co., LTD.) for sequencing, following the manufacturer's protocol. A total of 183 participants (112 NC and 71 CI) underwent APOE genotyping. The APOE ε4 carrier status was coded as "1″ for both homozygous and heterozygous carriers of ε4, and as "0″ for non-carriers.

### 2.7 Data analysis

All statistical analyses were performed using Python 3.8 and OriginPro 2021, with a p-value of <0.05 considered statistically significant. Differences in demographic characteristics, cognitive scores, and digital features were presented as medians with interquartile ranges. The Chi-square test was used to analyze gender differences between groups, and an independent sample *t*-test was applied to compare body mass index (BMI) when the data followed a normal distribution. For continuous variables that did not meet the assumptions of normality, the Mann-Whitney U test was used.

During the preprocessing phase, indicators with more than 10% missing data were excluded. Missing values in the remaining indicators were imputed using the multiple imputation method with default parameters. To address data imbalance, a random stratified downsampling method was applied to adjust the control dataset. Subsequently, the dataset was randomly split into a training set and a test set at a 7:3 ratio. In the model building phase, analysis of differences between groups was used to screen the meaningful features. The LightGBM algorithm was employed to construct machine learning diagnostic models for distinguishing patients with CI from NC, MCI from NC, and MCI from dementia. These algorithms were implemented using sklearn package.

Model performance was evaluated using receiver operating characteristic (ROC) curves, and key evaluation metrics included accuracy, sensitivity, specificity, and the area under the curve (AUC). SHAP (SHapley Additive exPlanations) values were utilized to interpret the contribution of individual features and their clinical relevance.

Considering the significant differences in demographic characteristics between groups, we developed models both with and without covariates, including age (years), sex (coded as "1″ for females and "2″ for males), education (years), and BMI (kg/m^2^). Pearson correlation analysis was conducted to explore the relationship between the top 10 key features of each digital tool and plasma p-tau217 levels, aiming to identify features most correlated with neuropathological changes in AD.

## 3 Result

### 3.1 Characteristic of study participants

The median age of the 1083 participants was 70 years (range: 65–93 years), and 55.2% were female. Among these participants, 337 were diagnosed with CI, including 216 MCI and 121 dementia, while the remaining 746 were classified as NC. Compared to NCs, patients with CI were older, more likely to be female, and had fewer years of education. Additionally, the AD8, Mini-Cog, MMSE, ADL, and CDR scores were significantly worse in patients with CI compared to NCs(*p<0.001*). There was no significant difference in the APOE ε4 carrier status between the two groups(*p=0.917*) (Table 1).

**Table 1:** Baseline demographic and clinical characteristics of study participants.

Characteristics	Total sample, (n=1083)	NC, (n=746)	CI, (n=337)	*p-*value
Age(years), M(IQR)	70(67, 74)	69(66, 73)	73(68, 77)	<0.001
Sex(Female), n(%)	598(55.2%)	396(53.1%)	202(59.9%)	0.036
Education(years), M(IQR)	7(5, 9)	8(6, 11)	6(2, 9)	<0.001
Primary or below(≤6 years)	495(45.7%)	279(37.4%)	216(64.1%)	/
Middle school or above (>6 years)	588(54.3%)	467(62.6%)	121(35.9%)	/
BMI(kg/m^2^), M(SD)	23.7(3.3)	23.9(3.2)	23.4(3.5)	0.04
AD8(score), M(IQR)	1(0, 2)	0(0, 1)	2(1, 4)	<0.001
Mini-Cog(score), M(IQR)	3(2, 5)	4(3, 5)	2(0, 3)	<0.001
MMSE(score), M(IQR)	24(19, 27)	27(24, 28)	20(15, 24)	<0.001
ADL(score), M(IQR)	22(20, 26)	20(20, 21)	25(22, 30)	<0.001
CDR(score), M(IQR)	0.5(0, 0.5)	0	0.5(0.5, 1)	<0.001
APOE ε4 allele, n/total(%)	29/183(15.8%)	18/112(16.1%)	11/71(15.5%)	0.917

*Note:* Date are median (IQR), mean (SD), or n (%). NC, normal control; CI, cognitive impairment; IQR, interquartile range; BMI, body mass index; AD8, Ascertain Dementia 8-item questionnaire; MMSE, Mini-Mental State Examination; ADL, Activity of Daily Living; CDR, Clinical Dementia Rating; APOE ε4, Apolipoprotein E eplison 4.

### 3.2 Differential features of digital screening tools between NC and CI groups

**Fig. 3:**
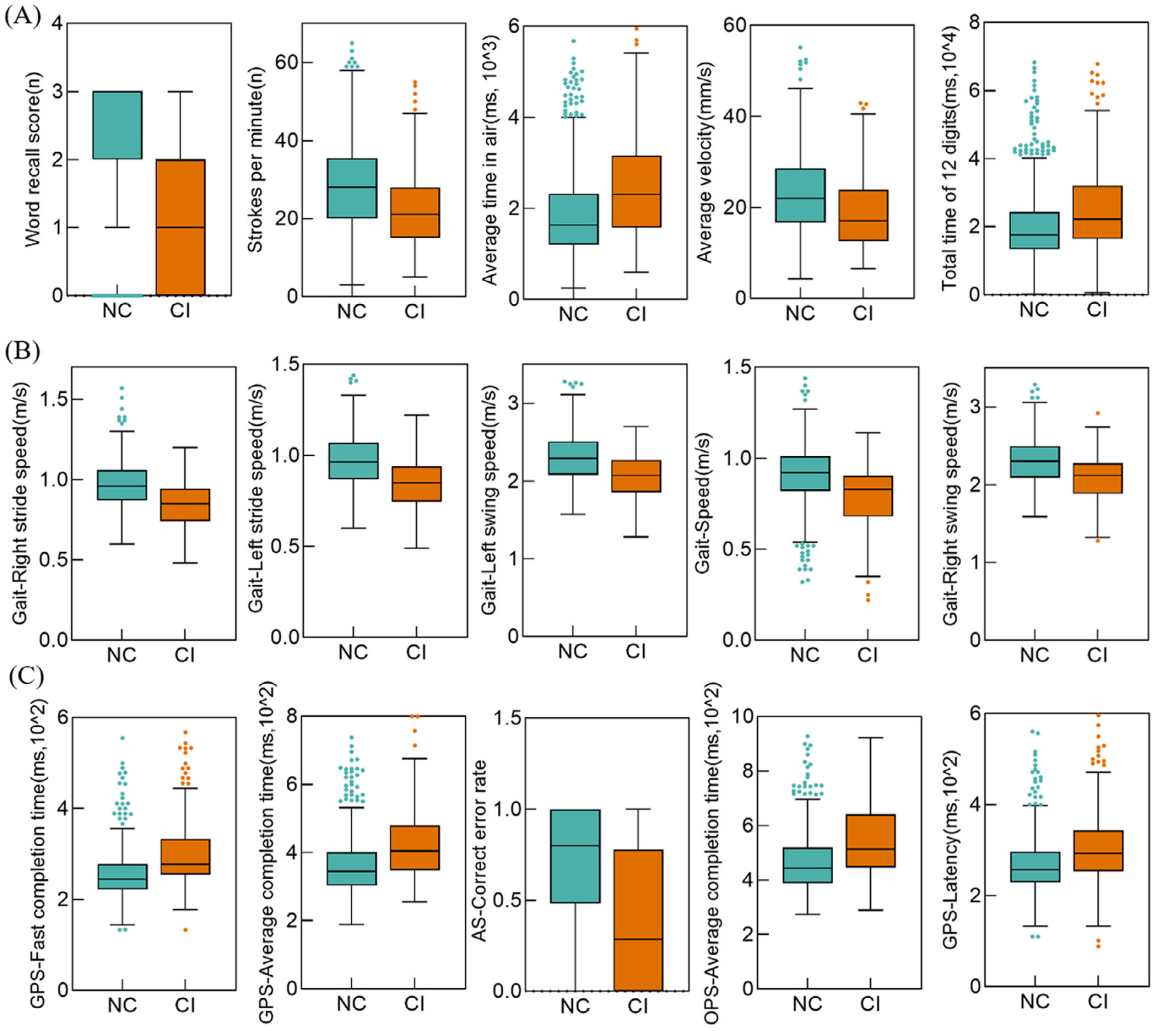
Box plots showing the top 5 features of digital screening tools with significantly different levels between NC and CI groups (all *p<0.001*). (A) Drawing test; (B) Gait test; (C) Eye tracking test. GPS, gap pro-saccade; OPS, overlap pro-saccade; AS, anti-saccade.

We extracted 28 drawing features, 125 gait features (including 50 DTC values), and 36 eye-tracking features. To identify features that significantly contributed to the classification models, we first analyzed inter-group differences between the NC and CI groups. Significant differences were observed in 21 drawing features, 71 gait features, and 35 eye-tracking features between the NC and CI groups (all p<0.05) (see Tables S6–S8 in the supplemental materials). The top five distinctive features from each digital screening tool are displayed in Fig. 3 (*all p<0.001*).

### 3.3 Effectiveness of digital tools and their fusion models in distinguishing CI from NC

**Fig. 4:**
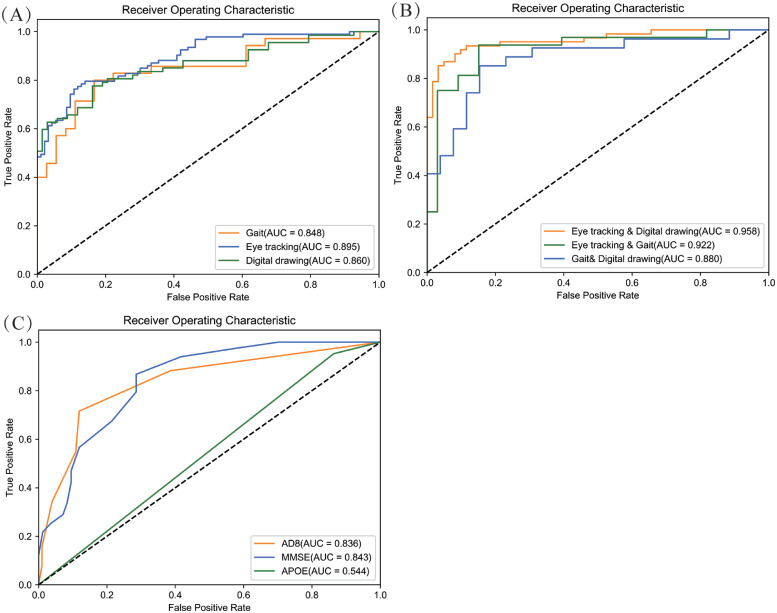
AUC comparison plots of digital screening tools and their fusion models in distinguishing CI from NC. (A) Drawing, gait, and eye-tracking models; (B) Fusion models based on two-paired tools; (C) Traditional screening models based on AD8, MMSE, and APOE genotyping. NC, normal control; CI, cognitive impairment; AD8, Ascertain Dementia 8-item questionnaire; MMSE, Mini-Mental State Examination; APOE, Apolipoprotein E; AUC, area under the curve.

We evaluated the effectiveness of these models in identifying patients with CI, compared to traditional screening tools (Fig. 4). In single models without covariates, the AUC for the drawing model was 0.860, while the AUC for the gait and eye-tracking models were 0.848 and 0.895, respectively (Fig. 4 A). The diagnostic performance of digital tools was equal to or better than that of traditional methods (AUC: AD8 = 0.836, MMSE = 0.843, APOE = 0.544, Fig. 4 C). Since the AD8 and MMSE were used as screening and diagnostic tools in the initial phase of the study, their effectiveness in classifying CI is expected to improve. Fig. 6 illustrates the impact of the top 10 features of each digital tool on the CI model's predictions.

Using pairwise combinations of digital tools, we developed three fusion models (Fig. 4 B). The model combining gait and drawing features achieved an AUC of 0.880. When eye-tracking and gait features were combined, the AUC increased to 0.922, with a sensitivity of 93.8%. The model based on eye-tracking and drawing features showed the best overall performance, with an AUC of 0.958, accuracy of 90.2%, sensitivity of 93.4%, and specificity of 86.9% (Table 2). The fusion models significantly improved the accuracy of CI detection, particularly the model combining eye-tracking and drawing features, which outperformed any single screening tool. The inclusion of covariates such as age, sex, education level, and BMI slightly reduced the AUC of each model, but these differences were not statistically significant (Table 2). The model combining eye-tracking and drawing features continued to show the highest AUC of 0.946.

**Table 2:** Diagnostic metrics of digital screening tools and their combined models in distinguishing CI/MCI from NC and MCI from dementia individuals.

Diagnostic models	AUC	Accuracy	Sensitivity	Specificity
337 NC vs. 337 CI (cognitive impairment) without covariates				
Digital drawing	0.860	0.770	0.687	0.853
Gait	0.848	0.789	0.800	0.778
Eye tracking	0.895	0.812	0.796	0.828
Eye tracking& Digital drawing	0.958	0.902	0.934	0.869
Eye tracking& Gait	0.922	0.877	0.938	0.818
Gait& Digital drawing	0.880	0.830	0.852	0.808
AD8	0.836	0.798	0.716	0.881
MMSE	0.843	0.731	0.675	0.786
APOE	0.544	0.488	1.000	0.000

*337 NC vs. 337 CI (cognitive impairment) with covariates*				
Digital drawing	0.846	0.758	0.682	0.833
Gait	0.820	0.783	0.853	0.714
Eye tracking	0.867	0.796	0.720	0.871
Eye tracking& Digital drawing	0.946	0.925	0.917	0.933
Eye tracking& Gait	0.892	0.825	0.839	0.812
Gait& Digital drawing	0.824	0.731	0.654	0.808
AD8	0.826	0.768	0.778	0.758
MMSE	0.841	0.761	0.716	0.805
APOE	0.632	0.595	0.619	0.571

*216 NC vs. 216 MCI (mild cognitive impairment) without covariates*				
Digital drawing	0.823	0.779	0.632	0.923
Eye tracking	0.872	0.780	0.722	0.836
Eye tracking& Digital drawing	0.928	0.862	0.781	0.939
p-tau217(50 *NC and 50 MCI)*	0.762	0.767	0.800	0.733
p-tau217& Eye tracking & Digital drawing (50 *NC and 50 MCI)*	0.942	0.833	0.867	0.800

*216 NC vs. 216 MCI (mild cognitive impairment) with covariates*				
Digital drawing	0.820	0.776	0.632	0.921
Eye tracking	0.864	0.797	0.757	0.838
Eye tracking & Digital drawing	0.922	0.816	0.760	0.875
p-tau217(50 *NC and 50 MCI)*	0.831	0.767	0.733	0.800
p-tau217& Eye tracking & Digital drawing(50 *NC and 50 MCI)*	0.929	0.833	0.867	0.800

*121 MCI vs. 121 Dementia without covariates*				
Digital drawing	0.562	0.531	0.562	0.500
Eye tracking	0.672	0.688	0.750	0.625
Eye tracking & Digital drawing	0.746	0.688	0.812	0.562

*121 MCI vs. 121 Dementia with covariates*				
Digital drawing	0.698	0.633	0.467	0.800
Eye tracking	0.747	0.700	0.867	0.533
Eye tracking & Digital drawing	0.796	0.667	0.533	0.800

Note: Covariates include age, sex, education level, and BMI. AUC, area under the curve; AD8, Ascertain Dementia 8-item questionnaire; MMSE, Mini-Mental State Examination; APOE, Apolipoprotein E; p-tau217, phosphorylated tau 217.

### 3.4 Effectiveness of distinguishing MCI from NC and dementia

**Fig. 5:**
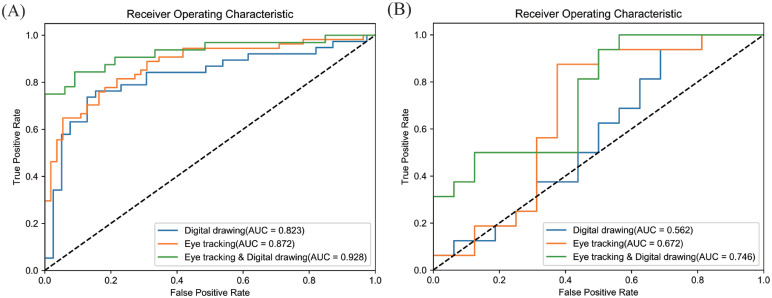
Power of the drawing-eye tracking fusion model in distinguishing MCI from NC and dementia. (A) For distinguishing MCI from NC, the drawing-eye tracking fusion model achieved the best AUC of 0.928; (B) For distinguishing MCI from dementia, the AUC of the drawing-eye tracking fusion model was significantly reduced to 0.746. NC, normal control; MCI, mild cognitive impairment; AUC, area under the curve.

Given the superior performance of the fusion model combining eye-tracking and drawing features, we focused on evaluating the effectiveness of these two digital tools in distinguishing MCI from NC and dementia. The fusion model combining eye-tracking and drawing features achieved the highest AUC for distinguishing MCI from NC (0.928) (Fig. 5 A). However, the AUC dropped to 0.746 when distinguishing MCI from dementia(Fig. 5 B), indicating that the fusion model should be applied with caution in patients with both MCI and dementia. The inclusion of covariates such as age, sex, education level, and BMI slightly reduced the AUC of each model, but the differences were not statistically significant (Table 2). The fusion model combining eye-tracking and drawing features still showed the highest AUC.

**Fig. 6:**
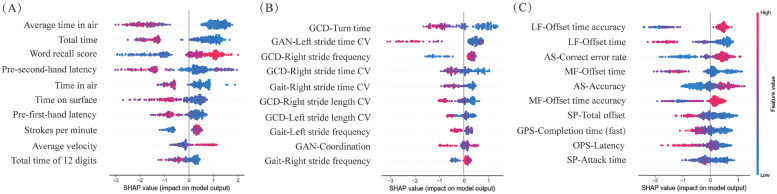
Description of SHapley Additive exPlanations (SHAP) values for CI detection models. The figure illustrates the impact of each feature on the model's predictions, ranked by their importance. The color gradient represents the range of feature values, from low (blue) to high (red). (A) Drawing model; (B) Gait model; (C) Eye tracking model. GCD, gait with counting down from 100; GAN, gait with animal naming; CV, coefficient of variation; SP, smooth pursuit; MF, median fixation; LF, Lateral fixation; GPS, gap pro-saccade; OPS, overlap pro-saccade; AS, anti-saccade.

### 3.5 Associations between key features and plasma p-tau217 levels

**Fig. 7:**
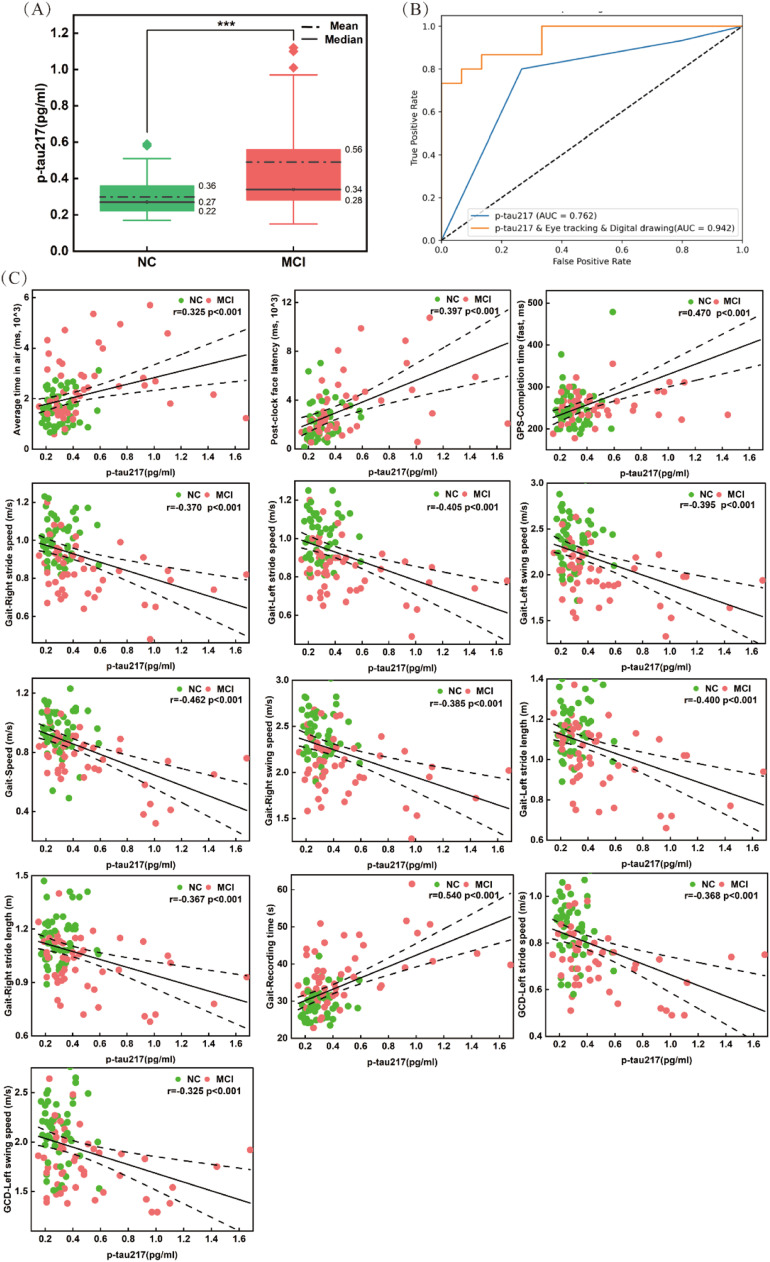
Comparison of plasma p-tau217 and the eye tracking-drawing fusion model in MCI and NC groups. (A) Plasma p-tau217 levels gradually increased from NC to MCI. (B) P-tau217 combined the drawing and eye tracking could distinguish MCI from NC, with the best ACU of 0.942; (C) Pearson correlation analysis between key digital features and plasma p-tau217 levels(all *|r|>0.3, p<0.001)*. ***p<0.001; GPS, gap pro-saccade; GCD, gait with counting down from 100.

To assess the significance of digital tools in detecting early CI and their correlation with established AD-related neuropathological markers, we measured plasma p-tau217 levels in 100 participants. Plasma p-tau217 levels were significantly higher in patients with MCI compared to NCs (Fig. 7 A, p<0.001). P-tau217 was then used to differentiate MCI from NC, achieving an AUC of 0.762. When combined with the eye-tracking and drawing fusion model, the diagnostic power significantly improved, reaching an AUC of 0.942 (Fig. 7 B). Further correlation analysis revealed that two of the top 10 drawing features, including "Average time in air" and "Post-clock face latency," were moderately correlated with plasma p-tau217 (|r|>0.3, p<0.001). Additionally, ten gait features-such as Gait-Right stride speed, Gait-Left stride speed, Gait-Left swing speed, Gait-Speed, Gait-Right swing speed, Gait-Left stride length, Gait-Right stride length, Gait-Recording time, GCD-Left stride speed, and GCD-Left swing speed-were moderately correlated with plasma p-tau217(*|r|>0.3, p<0.001*). And GPS-Fast completion time showed a moderate correlation with plasma p-tau217 (|r|=0.470, p<0.001) (Fig. 7C). Additional weaker correlations are presented in Table S9 in the supplementary materials. Our findings suggest that these digital features could serve as potential biomarkers for MCI.

## 4 Discussion

This study is the first to compare the effectiveness of multiple digital screening tools for detecting CI in a large-scale community setting and to analyze the association between digital features and plasma p-tau217 levels. We found that three digital tools—drawing(AUC = 0.860), gait (AUC = 0.848), and eye tracking(AUC = 0.895)—could effectively distinguish CI from NC individuals. The discriminative power of the fusion models was significantly enhanced, particularly for the model combining eye tracking and drawing (AUC =0.958), which achieved accuracy, sensitivity, and specificity, all exceeding 85%. Additionally, the fusion model demonstrated an AUC of 0.928 in distinguishing MCI from NC, which was much higher than those reported by traditional methods. Therefore, these digital tools are feasible for early detection of CI in the community settings. Moreover, multiple digital features were significantly correlated with plasma p-tau217 levels, suggesting that these tools could serve as potential digital biomarkers for patients with MCI.

Previous research primarily utilized the paper version of the Mini-Cog scale [55,56]. A study in the Chinese physical examination population indicated that the AUC of the Mini-Cog scale was 0.79 in distinguishing CI from NC individuals, with low accuracy [55]. In the past decade, several studies have digitally transformed the drawing scale [14,[57], [58], [59]], especially the Digital clock drawing(dCDT) model developed using machine learning or deep learning methods to extract more fine-grained performance features [22,60]. A meta-analysis showed that the sensitivity and specificity of the dCDT model for MCI screening were 0.86 and 0.92, respectively, which was significantly superior to paper-and-pencil CDT [57]. Based on the enhanced predictive value of digital transformation, we developed a "digital Mini-Cog" model and evaluated its performance in large-scale community CI detection. The results showed that the AUC of digital Mini-Cog was 0.860 for distinguishing CI from NC, and 0.823 for identifying MCI. Its performance is significantly improved. Numerous studies have shown that the AUC of plasma p-tau217 is more than 90% in predicting amyloid-positive individuals [61,62]. We found that the average time in air and post-clock face latency were significantly correlated with plasma p-tau217 levels, suggesting that these features are related to amyloid levels in the brain. A previous study in normal older adults also found that a poor clock composite score was correlated with a greater amyloid or tau burden [22]. Since p-tau217 was detected in community populations without a confirmed biomarker diagnosis, its effectiveness for identifying MCI was significantly lower than in clinical studies [63].

In this study, the AUC of the eye tracking model in distinguishing CI from NC was 0.895, and that for identifying MCI was 0.872. Song et al. [64] utilized various eye-tracking paradigms similar to ours to construct the logistics regression model, achieving an AUC of 0.823 for identifying MCI. Another study constructed a multi-classification model for AD, MCI, and normal individuals based on the LightGBM machine learning algorithm, and obtained an AUC of 0.824 and an accuracy of 65.2% [19]. Recently, a large-scale cohort study showed that the AUC of the eye-tracking model to distinguish CI from NC was 0.926, and its power was significantly improved [31]. This is similar to our findings. However, the AUC for distinguishing MCI from NC was only 0.742 (gait and eye tracking fusion model) [31], significantly lower than our result. This discrepancy may be attributed to the abnormal proportion of MCI (16%) and dementia (33%) cases in the study. We also found that the GPS-fast completion time was significantly correlated with plasma p-tau217 level, suggesting that eye tracking may be a digital marker of early dementia. Notably, the applicability of eye tracking was limited in low-culture older adults, with an AUC of 0.741 in distinguishing CI from NC(see supplemental material). Yuan et al. [34] developed a VR eye movement cognitive assessment tool and verified its ability to detect CI across different education levels, achieving an AUC of 0.88 for the low-culture group (≤6 years) and 0.94 for the high-culture group (>6 years). Since the paradigm type in their study differs from ours, comparing their effectiveness is controversial. We found that older adults with lower education had significant difficulties understanding the system instructions, which may relate to local language and cultural differences. Therefore, the applicability of eye tracking to different educational levels still needs further verification in larger-scale studies.

A cross-sectional study reported that the AUC for rhythm and variability (dual-task gait) in distinguishing AD from NC were 0.734 and 0.719, respectively [27]. Lin et al. [31] observed that the AUC for a single gait test in detecting CI was 0.544, whereas it increased to 0.798 after adding the additional cognitive task (100–3). It embodies the advantage of dual-task gait evaluation. Du et al. [65] discovered that DTC parameters were statistically significant in differentiating between NC and MCI groups. Consequently, this study constructed a fusion model based on single-task, dual-task gait, and DTC parameters. As expected, the fusion model achieved the optimal AUC of 0.848 in distinguishing CI from NC. The high diagnostic performance is also attributed to the upgraded equipment. The gait system in this study uses deep learning algorithms to identify key nodes of motion automatically and can comprehensively capture human kinematics features (including velocity, stride length, turn time, variability, swing length, coordination, rhythm, etc.). In addition, we found that the top 10 gait features were significantly correlated with plasma p-tau217, further emphasizing the strong association between gait and Aβ pathological changes. Previous studies have also found that the G3B-Turn time and DTC-Turn time are significantly correlated with plasma p-tau181, which may be related to the severity of CI [31]. Due to the small sample size, we did not verify the effectiveness of this model in distinguishing MCI from NC and dementia.

A previous study found that a fusion model based on gait and drawing features achieved an accuracy of 86.1% and an AUC of 0.93 in classifying AD, MCI, and NC [43]. Similarly, Lin et al. [31] reported that the AUC for the dual-task eye tracking and gait fusion model in distinguishing CI from NC was 0.987. These findings are consistent with our results. We observed that combining multiple behavioral tools significantly enhanced classification effectiveness compared to using any single tool. Specifically, the AUC for the eye tracking and drawing fusion model was 0.958 in distinguishing CI from NC. This enhanced diagnostic effectiveness is partly attributed to the heterogeneity of patients with CI, which cannot be fully explained by a single pathological process [66]. Moreover, different behavioral features provide distinct and complementary cognitive insights, enabling a more comprehensive understanding of the underlying pathology [20,39,67]. Although the accuracy of these models does not yet surpass that of traditional examinations(cerebrospinal fluid and PET) [62,68], it is significantly better than traditional tests. Additionally, considering the significant effects of age on cognition, gait, and eye-tracking performance [69], we incorporated demographic characteristics into each classification model but found that the increase in AUC was not significant.

These digital screening tools have demonstrated good diagnostic validity; however, their cost-effectiveness for large-scale deployment remains unclear. The digital drawing test is cost-effective, requiring only a tablet device, making it widely accessible. In contrast, gait and eye-tracking devices are relatively expensive and have higher costs. Despite this, these tools offer significant advantages in community-based large-scale cognitive screening, such as simplicity of operation and automated scoring. They can be administered by non-specialist staff who guide participants through the tests, and automated scoring reduces the time and effort required for professional training. A study on non-clinician testers found that 5% of traditional tests had administrative errors, and 32% had scoring errors, highlighting the need to retrain testers every 2 to 3 months [70]. Moreover, a recent unsupervised digital speech test reduced testing time by 16.2% to 36% compared to the MMSE or MoCA test, achieving a completion rate of 97.5% [71]. Additionally, the gait devices used in this study do not require wearable sensors, saving substantial time during screening and significantly improving efficiency. In the context of global efforts to prevent and control dementia, community or primary care centers serve as the frontline for CI screening. There is an urgent need for high-efficiency and reliable digital tools that can be operated without highly trained professionals, minimizing the waste of healthcare resources. However, we also found that these digital screening tools may not be suitable for patients with severe CI, as they may be unable to complete the tests or lack sufficient digital literacy.

In addition, a notable finding is the relatively low prevalence of the APOE ε4 allele in our study, measured at 15.8%. A previous study examining APOE genotypes in northern Chinese populations reported a prevalence of 25% for the APOE ε4 allele [72]. However, participants in that study were recruited through advertising and exhibited constraints in age and education levels. Another study, involving older adults from rural Chinese communities, reported a similar prevalence of 15.87% for the APOE ε4 allele, aligning with our findings [73]. Additionally, a study investigating the APOE genotype in older African-American and Caucasian community residents identified the APOE ε4 allele in 29% of the population [74]. A study on genetic factors associated with AD in older adults from the Israeli Arab community reported the world's lowest prevalence of the APOE ε4 allele, at 6.3% [75]. These findings suggest that the prevalence of the APOE ε4 allele varies significantly across ethnicities, geographical regions, and age groups. This variability may be influenced by global genetic patterns, local ancestral traits, and gene-environment interactions.

However, our study has several limitations. First, this study used the AD8 combined with the Mini-Cog scale for baseline cognitive screening (a parallel test, where a positive result in either test indicates a risk for CI). Individuals identified as at risk for CI were further evaluated to establish an objective CI diagnosis. Previous studies have found that this parallel test demonstrates good efficacy in detecting CI or dementia. However, its performance in identifying MCI remains unclear. A small-sample study reported that the parallel test achieved an AUC of 0.76 and a sensitivity of 90.91% for detecting CI in a Chinese health screening population [55]. Additionally, a large-scale community screening study in eastern China found that the parallel test had an AUC of 0.84 and a sensitivity of 95.72% for detecting dementia [56]. These findings suggest that the parallel test may be more suitable for detecting advanced CI. As such, our study may have missed some cases of MCI, as many researchers have also raised concerns. Therefore, caution should be exercised when using the AD8 combined with the Mini-Cog scale as a screening tool for CI in community settings, particularly for identifying MCI. Second, the reliability and validity of the Mini-Cog scale in the Chinese population have not yet been established. We translated and adapted the original Mini-Cog scale to better align with local cultural norms (see supplementary materials). The applicability and optimal cutoff values will be explored in future publications. Third, no neuroimaging techniques (*e.g.*, brain MRI or CT) were performed in this study, making it difficult to determine the exact etiology of CI. Finally, the small sample sizes for plasma p-tau217 and gait tests may limit the reliability of the findings, especially for gait testing. Our study lacks evidence on the ability of gait tests to detect MCI among NC individuals.

In conclusion, our findings support the use of digital tools as effective methods for the early detection of CI in community settings and as potential digital markers for AD. Future research should focus on longitudinal analyses and explore the association between digital features and biomarkers derived from imaging and PET scans. Ultimately, the goal is to develop a universally accessible, highly accurate diagnostic toolkit that can be implemented in community health settings worldwide, facilitating earlier and more effective interventions.

## Consent statement

All participants provided written informed consent (electronic version).

## Declaration of generative AI and AI-assisted technologies in the writing process

The authors declared that they did not use the AI and AI-assisted technologies in the writing process.

## Funding sources

This work was supported by the Surface Project of Natural Science Research of Shanxi Province [grant number 202203021221255]; the Youth Project of Applied Basic Research Project of Shanxi Province [grant number 202203021212042]; and the 10.13039/501100001809National Natural Science Foundation Project [grant number U21A20386].

## CRediT authorship contribution statement

**Xiaonan Zhang:** Writing – original draft. **Feifei Zhang:** Writing – original draft, Investigation. **Sijia Hou:** Investigation, Data curation. **Chenxi Hao:** Project administration, Methodology. **Xiangmin Fan:** Software, Methodology, Formal analysis. **Yarong Zhao:** Project administration, Investigation, Data curation. **Wenjing Bao:** Resources, Investigation, Data curation. **Junpin An:** Resources, Methodology, Investigation, Data curation. **Shuning Du:** Investigation, Data curation. **Guowen Min:** Data curation, Conceptualization. **Qiuyan Wang:** Resources, Project administration, Investigation, Conceptualization. **Wencheng Zhu:** Visualization, Validation, Supervision, Software. **Yang Li:** Writing – review & editing, Supervision, Project administration, Funding acquisition. **Hui Zhang:** Writing – review & editing, Project administration, Conceptualization.

## Declaration of competing interest

The authors declare that they have no conflicts of interest.

## Appendix Supplementary materials

Image, application 1
