# Supplementary material for: Effectiveness of digital screening tools in detecting cognitive impairment among community-dwelling elderly in Northern China: A large cohort study
Source: J Prev Alzheimers Dis. 2025 Feb 7;12(3):100080. doi: 10.1016/j.tjpad.2025.100080 (PMC12184026; doi:10.1016/j.tjpad.2025.100080)
Supplement: Supplementary file 1 [file mmc1.docx]

**Supplementary materials**


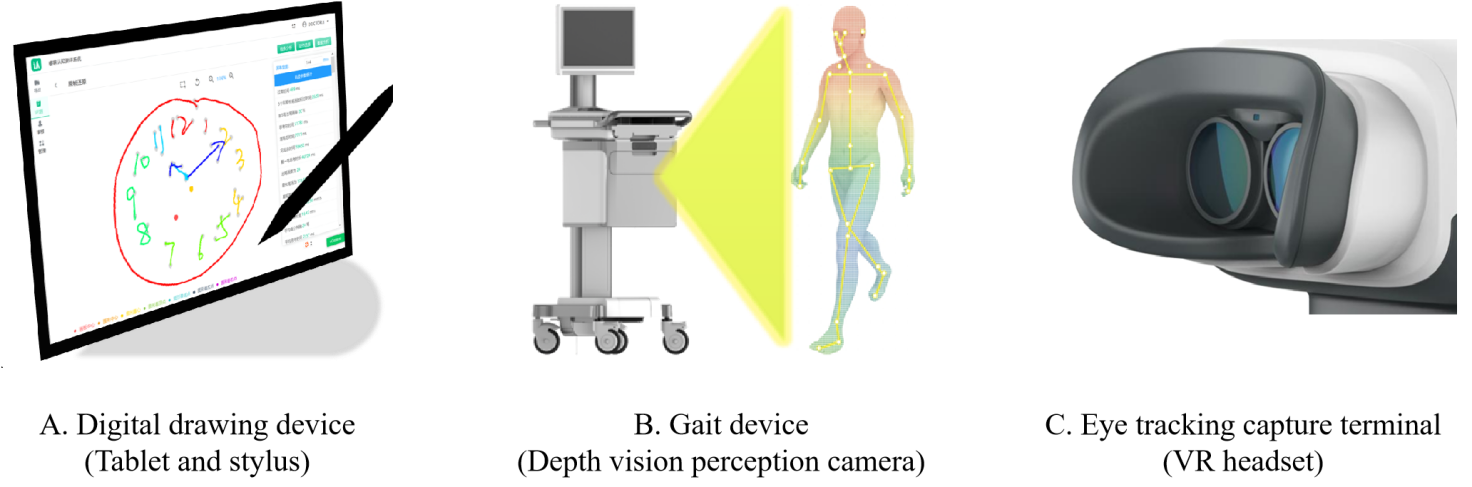


**Fig. S1** Schematic diagram of three digital screening tools

**Digital drawing test**

Word memory subtest

**Table S1** Six versions of the word list in the word memory subtest

| Version 1 | Version 2 | Version 3 | Version 4 | Version 5 | Version 6 |
| --- | --- | --- | --- | --- | --- |
| 香蕉 | 领导 | 山脉 | 河流 | 船长 | 女儿 |
| 日出 | 季节 | 厨房 | 国家 | 公园 | 天堂 |
| 椅子 | 桌子 | 宝宝 | 手指 | 图片 | 山峰 |

Clock drawing subtest (CDT)

CDT is jointly designed and developed by the First Hospital of Shanxi Medical University and Shanxi Infreisi Technology Co., LTD, which holds the license for this study. Developed from the traditional pen-and-paper test, the digital CDT is administered on a 10.8-inch Huawei Tablet M6, featuring a resolution of 2560×1600 and equipped with a stylus. Participants complete the test using the stylus, and the device captures a series of time-stamped (x, y) coordinates at a 60 Hz sampling frequency. It can extract multiple objective parameters about the drawing process and reproduce the drawing trajectory. The CDT device extracts feature parameters in two steps: First, the human observer manually classifies each stroke into clock components (i.e., clock face, hands, numbers, noise). Then, the digital device can automatically analyze and extract 27 feature parameters, including time in air, time on surface, total time, average time in air, post-clock face latency, clock face residence, pre-first-hand latency, pre-second-hand latency, pre-first-stroke latency, total strokes, longest stroke length, shortest stroke length, longest stroke velocity, average length, strokes per minute, average velocity, clock face area, minute hand length, minute hand velocity, hour hand length, hour hand velocity, minute residence, hour residence, minute/hour hand rate, total time in twelve digits, integrity of twelve digits , and accuracy of twelve digits. These fine-grained parameters provide additional details about performance.

The optimal algorithm rules of the Mini-Cog scale are as follows: (1) subjects recalling none of the three words were classified as dementia; (2) subjects recalling all three words were classified as non-dementia; (3) subjects recalling intermediate words (1~2) were classified based on results of the CDT (abnormal = dementia, normal = non-dementia). A Mini-Cog score <3 was considered as abnormal cognition.

**Table S2** The definition of digital drawing parameters

| Parameters | Definition |
| --- | --- |
| Time in air | Pen-up state, total time while moving the pen from one stroke to the next, ms |
| Time on surface | Pen-down state, total time of each stroke staying on the tablet, ms |
| Total time | The sum of time in air and time on the surface, ms |
| Average time in air | The ratio of time in air to total strokes, ms |
| Post-clock face latency | Time taken between completing the clock face and drawing the first number, ms |
| Clock face residence | Total time from beginning to end of drawing the clock face, ms |
| Pre-first-hand latency | Time taken between drawing the first hand and the previous stroke, ms |
| Pre-second-hand latency | Time taken between drawing the second hand and the previous stroke, ms |
| Pre-first-stroke latency | Time taken between the start and the first stroke, ms |
| Total strokes | / |
| Longest stroke length | / |
| Shortest stroke length | / |
| Longest stroke velocity | The average velocity of the longest stroke, mm/s |
| Average length | The ratio of total stroke length to total number, mm |
| Strokes per minute | The ratio of total strokes to total time multiplied by 60, n |
| Average velocity | The ratio of total time to total number, mm/s |
| Clock face area | the minimum circumscribed circle area of the figure(πr^2^), where r is the average of the horizontal and vertical radii, cm^2^ |
| Minute hand length | / |
| Minute hand velocity | The average velocity of the minute hand, mm/s |
| Hour hand length | / |
| Hour hand velocity | The average velocity of the hour hand, mm/s |
| Minute residence | Time of drawing minute hand on the tablet, ms |
| Hour residence | Time of drawing hour hand on the tablet, ms |
| Minute/hour hand rate | The ratio of minute hand length to hour hand length, % |
| Total time in twelve digits | total time between drawing the first digit and the last digit, ms |
| Integrity of twelve digits | the percentage of correct digits in 12 digits, regardless of digit position, % |
| Accuracy of twelve digits | the percentage of correct digits in 12 digits, and the digit position must be correct, % |

**Table S3** The definition of eye tracking parameters

| Parameters | Definition |
| --- | --- |
| Offset number | the number of the gaze point deviating from the center of the target by more than 4°, n |
| Total offset (4°) | the total offset of the gaze point deviating from the center of the target by more than 4°, % |
| Saccade accuracy | the ratio of the number of successful saccades to the total number of completed saccades, % |
| Saccade latency | the duration between the start of the target and the start of the saccade, ms |
| Saccade speed | the angular speed of eye movement in the time interval between saccade onset and offset, °/s |
| Saccade total time | the duration of saccade completion, ms |
| Anti-saccade accuracy | the ratio of the number of saccades in the opposite direction to the number of completed saccades, % |
| Anti-saccade error correction rate | the ratio of the number of subsequent corrections to the total number of errors in a patient's failure to suppress saccades in the target direction, % |

**Table S4** Demographic of patients with cognitive impairment

| Characteristics | MCI(n=216) | Dementia(n=121) | *p-*value |
| --- | --- | --- | --- |
| Age(years), M(IQR) | 72(67, 76) | 74(70, 81) | <0.001 |
| Sex(Female), n(%) | 130(60.2%) | 72(59.5%) | 0.903 |
| Education(years), M(IQR) | 6(3, 9) | 5(0, 9) | 0.178 |
| BMI(kg/m^2^), M(SD) | 23.7(3.5) | 23.0(3.6) | 0.09 |

**Table S5** Demographic of participants for plasma p-tau217 assay

| Characteristics | NC(n=50) | MCI(n=50) | *p-*value |
| --- | --- | --- | --- |
| Age(years), M(IQR) | 69(67, 72) | 73(69, 80) | 0.001 |
| Sex(Female), n(%) | 32(64%) | 33(66%) | 0.834 |
| Education(years), M(IQR) | 12(9, 12) | 7.5(5, 9) | <0.001 |
| BMI(kg/m^2^), M(SD) | 23.5(2.6) | 24.4(3.5) | 0.151 |

**Table S6** Difference parameters of drawing test between NC and CI groups (*p*<0.05)

| Parameters | NC (n=665) | CI (n=225) | *p-*value |
| --- | --- | --- | --- |
| Post-clock face latency (ms) | 1953 (1227.5, 3419) | 2485 (1559, 3973) | <0.001 |
| Pre-first-hand latency (ms) | 5136 (2925.5, 8571.5) | 6885 (3852.5, 11144) | <0.001 |
| Pre-second-hand latency (ms) | 1547 (762.5, 3071) | 2102 (1158, 4445) | <0.001 |
| Minute hand length (mm) | 11.68 (8.36, 15.31) | 10.45 (7.22, 14.2) | 0.011 |
| Minute hand velocity (mm/s) | 12.82 (8.38, 18.4) | 10.64 (6.87, 15.15) | <0.001 |
| Total time of 12 digits (ms) | 17256 (13268, 24143.5) | 21415 (15638, 30387) | <0.001 |
| Integrity of 12 digits (%) | 100 (100, 100) | 100 (100, 100) | 0.019 |
| Accuracy of 12 digits (%) | 100 (91.6, 100) | 100 (66.67, 100) | <0.001 |
| Time in air (ms) | 37094 (26048, 55543) | 48222 (33088.5, 69173.5) | <0.001 |
| Time on surface (ms) | 11661 (9412, 14789.5) | 14121 (10375.5, 17317) | <0.001 |
| Total time (ms) | 49416 (36584, 70367.5) | 61898 (45703.5, 90715.5) | <0.001 |
| Pre-first-stroke latency (ms) | 10433 (6171.5, 17885.5) | 14582 (8432, 23533.5) | <0.001 |
| Longest stroke length (mm) | 107.21 (83.64, 131.87) | 97.03 (70.49, 127.37) | 0.005 |
| Shortest stroke length (mm) | 1.41 (0.57, 2.33) | 1.06 (0.25, 1.97) | <0.001 |
| Longest stroke velocity (mm/s) | 49.45 (34.8, 65.51) | 37.34 (28.02, 56.8) | <0.001 |
| Average length (mm) | 11.49 (9.18, 14) | 10.79 (8.48, 13.13) | 0.013 |
| Strokes per minute (n) | 28 (21, 37) | 22 (16, 29) | <0.001 |
| Average time in air (ms) | 1614 (1176.5, 2318.5) | 2154 (1503.5, 2923) | <0.001 |
| Average velocity (mm/s) | 22.08 (16.92, 28.8) | 16.68 (12.66, 24) | <0.001 |
| Clock face area (mm²) | 928.33 (573.83, 1383.46) | 770.43 (456.93, 1216.75) | <0.001 |
| Word recall score | 3 (2, 3) | 1 (0, 2) | <0.001 |

*Note:* NC, normal control; CI, cognitive impairment; ms millisecond; mm, millimeter; s, second; n, number; mm², square milimeter.

**Table S7** Difference parameters of gait test between NC and CI groups (*p*<0.05)

| Parameters | NC(n=418) | CI(n=117) | *p-*value |
| --- | --- | --- | --- |
| Gait-Recording time (s) | 31.37(27.94, 35.68) | 34.67(31.45, 42.04) | <0.001 |
| Gait-Left stance (%) | 67.85(67.11, 68.75) | 68.57(67.56, 69.51) | 0.004 |
| Gait-Right stance (%) | 67.64(66.66, 68.57) | 68.57(67.64, 69.44) | <0.001 |
| Gait-Left swing (%) | 32.14(31.25, 32.88) | 31.42(30.49, 32.43) | 0.004 |
| Gait-Right swing (%) | 32.35(31.42, 33.33) | 31.42(30.55, 32.35) | <0.001 |
| Gait-Left double support (%) | 36.17(34.83, 37.83) | 37.29(36.11, 38.88) | <0.001 |
| Gait-Right double support (%) | 35.86(34.37, 37.5) | 37.17(35.69, 38.8) | <0.001 |
| Gait-Left stride length (m) | 1.11(1.02, 1.19) | 1.02(0.92, 1.12) | <0.001 |
| Gait-Right stride length (m) | 1.12(1.02, 1.19) | 1.01(0.91, 1.13) | <0.001 |
| Gait-Left step height (m) | 0.12(0.11, 0.14) | 0.12(0.1, 0.13) | 0.037 |
| Gait-Right step height (m) | 0.12(0.11, 0.14) | 0.12(0.1, 0.13) | 0.013 |
| Gait-Speed (m/s) | 0.92(0.82, 1.01) | 0.82(0.67, 0.9) | <0.001 |
| Gait-Stride frequency (steps/min) | 105.88(97.36, 112.49) | 100(94.73, 105.88) | 0.006 |
| Gait-Left stride speed (m/s) | 0.96(0.87, 1.07) | 0.85(0.75, 0.94) | <0.001 |
| Gait-Right stride speed(m/s) | 0.96(0.87, 1.06) | 0.85(0.74, 0.95) | <0.001 |
| Gait-Left swing speed (m/s) | 2.29(2.07, 2.51) | 2.07(1.86, 2.27) | <0.001 |
| Gait-Right swing speed(m/s) | 2.3(2.09, 2.5) | 2.12(1.89, 2.28) | <0.001 |
| Gait-Turn time (s) | 1.5(1.3, 1.8) | 1.76(1.43, 2.25) | <0.001 |
| Gait-Coordination | 0(0, 4.87) | 0(0, 0) | 0.001 |
| Gait-Right stride length CV | 4.27(3.29, 5.48) | 4.45(3.68, 5.96) | 0.022 |
| GCD-Recording time (s) | 36.25(30.59, 42.57) | 39.99(34.03, 51.3) | <0.001 |
| GCD-Left stance (%) | 68.75(67.64, 70) | 69.44(68.09, 70.73) | 0.012 |
| GCD-Right stance(%) | 68.75(67.64, 70.26) | 69.44(68.24, 70.83) | 0.005 |
| GCD-Left swing (%) | 31.25(30, 32.35) | 30.55(29.26, 31.91) | 0.015 |
| GCD-Right swing (%) | 31.25(29.75, 32.35) | 30.55(29.17, 31.75) | 0.005 |
| GCD-Left double support (%) | 37.5(35.82, 40) | 38.56(36.59, 41.38) | 0.001 |
| GCD-Right double support(%) | 37.5(35.67, 40) | 38.46(36.53, 41.06) | 0.003 |
| GCD-Stride width (m) | 0.13(0.12, 0.14) | 0.13(0.12, 0.15) | 0.014 |
| GCD-Left stride length (m) | 1.07(0.98, 1.17) | 1(0.85, 1.13) | <0.001 |
| GCD-Right stride length(m) | 1.07(0.97, 1.17) | 0.99(0.85, 1.12) | <0.001 |
| GCD-Left step height (m) | 0.12(0.1, 0.13) | 0.11(0.1, 0.13) | 0.006 |
| GCD-Right step height(m) | 0.11(0.1, 0.13) | 0.11(0.09, 0.13) | 0.035 |
| GCD-Speed (m/s) | 0.79(0.69, 0.92) | 0.7(0.58, 0.83) | <0.001 |
| GCD-Right stride frequency(steps/min) | 94.73(85.71, 100) | 90(78.26, 100) | 0.04 |
| GCD-Left stride speed (m/s) | 0.83(0.73, 0.96) | 0.73(0.64, 0.85) | <0.001 |
| GCD-Right stride speed (m/s) | 0.82(0.72, 0.95) | 0.74(0.64, 0.86) | <0.001 |
| GCD-Left swing speed (m/s) | 2.02(1.78, 2.25) | 1.83(1.54, 2.06) | <0.001 |
| GCD-Right swing speed(m/s) | 2.03(1.81, 2.29) | 1.85(1.62, 2.09) | <0.001 |
| GCD-Turn time (s) | 1.53(1.31, 1.84) | 1.8(1.48, 2.16) | <0.001 |
| GCD-Coordination | 0(0, 7.21) | 0(0, 6.45) | 0.019 |
| GAN-Recording time (s) | 42.72(34.34, 52.87) | 45.53(36.33, 56.51) | 0.032 |
| GAN-Stride width (m) | 0.13(0.12, 0.15) | 0.14(0.13, 0.15) | 0.002 |
| GAN-Left stride length (m) | 1.03(0.93, 1.14) | 0.96(0.83, 1.06) | <0.001 |
| GAN-Right stride length (m) | 1.03(0.93, 1.13) | 0.96(0.82, 1.07) | <0.001 |
| GAN-Left step height (m) | 0.11(0.1, 0.13) | 0.11(0.09, 0.12) | 0.03 |
| GAN-Right step height (m) | 0.11(0.1, 0.13) | 0.1(0.09, 0.12) | 0.024 |
| GAN-Speed (m/s) | 0.69(0.57, 0.84) | 0.66(0.55, 0.78) | 0.046 |
| GAN-Left stride speed (m/s) | 0.73(0.62, 0.86) | 0.67(0.56, 0.82) | 0.007 |
| GAN-Right stride speed (m/s) | 0.73(0.62, 0.86) | 0.68(0.59, 0.82) | 0.028 |
| GAN-Left swing speed (m/s) | 1.8(1.58, 2.08) | 1.7(1.53, 1.96) | 0.014 |
| GAN-Right swing speed (m/s) | 1.84(1.61, 2.11) | 1.78(1.53, 2.01) | 0.033 |
| GAN-Turn time (s) | 1.71(1.46, 2.23) | 1.9(1.6, 2.5) | <0.001 |
| GAN-Coordination | 3.38(0, 7.99) | 0(0, 6.67) | <0.001 |
| GAN-Right stride length CV | 7.66(4.95, 12.9) | 6.27(4.52, 9.7) | 0.035 |
| DTC_GCD-Speed (m/s) | 0.11(0.04, 0.18) | 0.08(-0.02, 0.18) | 0.028 |
| DTC_GAN-Recording time (s) | -0.25(-0.55, -0.13) | -0.2(-0.5, -0.02) | 0.012 |
| DTC_GAN-Right stance (%) | -0.03(-0.05, -0.01) | -0.02(-0.04, -0.01) | 0.003 |
| DTC_GAN-Right swing (%) | 0.06(0.02, 0.11) | 0.05(0.02, 0.09) | 0.005 |
| DTC_GAN-Right double support (%) | -0.06(-0.13, -0.03) | -0.06(-0.13, -0.01) | 0.006 |
| DTC_GAN-Right double support (%) | -0.09(-0.15, -0.04) | -0.06(-0.17, -0.02) | 0.002 |
| DTC_GAN-Stride width (m) | 0(-0.07, 0.07) | 0(-0.12, 0) | 0.007 |
| DTC_GAN-Left stride length (m) | 0.07(0.02, 0.12) | 0.05(-0.01, 0.1) | 0.044 |
| DTC_GAN-Speed (m/s) | 0.19(0.12, 0.31) | 0.12(0, 0.31) | <0.001 |
| DTC_GAN-Left stride frequency (steps/min) | 0.14(0.06, 0.22) | 0.08(0.05, 0.22) | 0.005 |
| DTC_GAN-Right stride frequency (steps/min) | 0.16(0.08, 0.24) | 0.1(0.04, 0.21) | <0.001 |
| DTC_GAN-Left stride speed (m/s) | 0.2(0.14, 0.3) | 0.14(0.04, 0.34) | <0.001 |
| DTC_GAN-Right stride speed (m/s) | 0.22(0.13, 0.32) | 0.14(0.07, 0.31) | <0.001 |
| DTC_GAN-Left swing speed (m/s) | 0.19(0.11, 0.26) | 0.12(0.03, 0.26) | <0.001 |
| DTC_GAN-Right swing speed (m/s) | 0.18(0.1, 0.25) | 0.12(0.02, 0.23) | <0.001 |
| DTC_GAN-Turn time (s) | -0.16(-0.46, 0.06) | -0.09(-0.51, 0.1) | 0.049 |
| DTC_GAN-Right stride length CV | -0.68(-1.88, -0.08) | -0.18(-0.79, 0.17) | <0.001 |

*Note:* NC, normal control; CI, cognitive impairment; GCD, gait with counting down from 100; GAN, gait with animal naming; DTC, dual-task cost, calculated as [(single task- dual task)/single task]×100%; CV, coefficient of variation, calculated by dividing the standard variability to mean value; s, second; %, percentage; m, meter; min, minute.

**Table S8** Difference parameters of eye tracking test between NC and CI groups (*p*<0.05)

| Parameters | NC(n=685) | CI(n=316) | *p*-value |
| --- | --- | --- | --- |
| SP-Attack time (ms) | 554.38(387.26, 734.36) | 619.76(437.56, 856.05) | 0.002 |
| SP-Tracking speed (°/s) | 19.72(16.5, 24.41) | 18.5(14.87, 23.18) | 0.013 |
| SP-Tracking acceleration (°/s²) | 37.35(23.83, 64.26) | 30.86(19.1, 57.68) | 0.011 |
| SP-Offset number (n) | 21(13, 34) | 26(16, 39.25) | <0.001 |
| SP-Total offset (>4°, °) | 112.47(62.57, 181.25) | 136.05(85.07, 213.77) | <0.001 |
| MF-Offset number (>4°, n) | 7(3, 14) | 9.5(5, 20) | <0.001 |
| MF-Offset number (>2°, n) | 21(10, 34) | 26(16, 39) | 0.001 |
| MF-Total offset (>4°, °) | 44.95(18.25, 90.11) | 64.72(30.85, 115.29) | <0.001 |
| MF-Offset time (<4°, ms) | 776.69(454.96, 1467.31) | 1009.7(621.4, 1916.95) | <0.001 |
| MF-Offset time accuracy (<4°, %) | 92.22(85.3, 95.44) | 89.89(80.81, 93.78) | <0.001 |
| LF-Offset number (>4°, n) | 27(15, 44.25) | 33(20, 56) | <0.001 |
| LF-Offset number (>2°, n) | 54(36, 77) | 62(43, 84.25) | 0.038 |
| LF-Total offset (>4°, °) | 204.18(139.94, 302.4) | 242.35(163.22, 363.54) | <0.001 |
| LF-Offset time (<4°, ms) | 4934.84(2787.43, 8714.93) | 6489.45(3999.82, 12200.49) | <0.001 |
| LF-Offset time accuracy (<4°, %) | 83.58(70.99, 90.72) | 78.4(59.39, 86.68) | <0.001 |
| OPS-Accuracy (%) | 100(83.33, 100) | 90(70, 100) | <0.001 |
| OPS-Latency (ms) | 365.78(317.55, 426.85) | 426.6(357.36, 497.55) | <0.001 |
| OPS-Completion time (fast, ms) | 300.35(266.56, 358.79) | 350.12(299.45, 433.5) | <0.001 |
| OPS-Completion time (average, ms) | 442.8(389.9, 512.72) | 512.05(448.72, 636.71) | <0.001 |
| OPS-Average speed (°/s) | 259.47(205.21, 309.57) | 220.88(171.4, 287.58) | <0.001 |
| OPS-Fastest speed (°/s) | 445.42(373.8, 544.59) | 402.72(337.79, 495.31) | <0.001 |
| GPS-Accuracy (%) | 100(85.71, 100) | 95(71.43, 100) | <0.001 |
| GPS-Latency (ms) | 257.67(229.99, 296.59) | 294.25(254.3, 345.85) | <0.001 |
| GPS-Completion time (fast, ms) | 244.68(222.07, 277.66) | 277.61(245.22, 322.56) | <0.001 |
| GPS-Completion time (average, ms) | 344.59(303.29, 398.28) | 402.37(344.68, 478.09) | <0.001 |
| GPS-Average speed (°/s) | 235.6(183.75, 284.96) | 206.52(147.56, 272.16) | <0.001 |
| GPS-Fastest speed (°/s) | 430.54(356.26, 535.22) | 379.72(314.41, 485.12) | <0.001 |
| AS-Accuracy (%) | 25(0, 44.44) | 0(0, 28.57) | <0.001 |
| AS-Latency (ms) | 333.1(287.71, 391.48) | 360.36(302.59, 426.04) | <0.001 |
| AS-Completion time (fast, ms) | 300.09(266.39, 343.99) | 327.35(288.55, 392.32) | <0.001 |
| AS-Completion time (average, ms) | 447.05(381.37, 512.51) | 484.65(417.2, 566.59) | <0.001 |
| AS-Correct error rate (%) | 80(50, 100) | 33.33(0, 80) | <0.001 |
| AS-Correct error time (average, ms) | 340.70(282.84, 428.46) | 425.79(323.58, 538.91) | <0.001 |
| AS-Average speed (°/s) | 236.96(184.28, 292.52) | 209.11(143.94, 267.36) | <0.001 |
| AS-Fastest speed (°/s) | 437.95(352.35, 543.8) | 394.79(298.41, 494.86) | <0.001 |

*Note:* NC, normal control; CI, cognitive impairment; SP, smooth pursuit; MF, median fixation; LF, Lateral fixation; PS, pro-saccade; GPS, gap pro-saccade; OPS, overlap pro-saccade; AS, anti-saccade; ms, millisecond; s, second; n, number.

**Table S9** Pearson correlation analysis between plasma p-tau217 and digital features

| Digital parameters | p-tau217 | |
| --- | --- | --- |
|  | *r-*value | *p-*value |
| Drawing | | |
| Word recall score | -0.294 | 0.003 |
| Strokes per minute | -0.254 | 0.011 |
| Average time in air | 0.325 | <0.001 |
| Average velocity | -0.275 | 0.006 |
| Total time of 12 digits | 0.140 | 0.165 |
| Total time | 0.277 | 0.005 |
| Time in air | 0.284 | 0.004 |
| Accuracy of 12 digits | -0.027 | 0.787 |
| Time on surface | 0.116 | 0.249 |
| Post-clock face latency | 0.397 | <0.001 |
| Gait | | |
| Gait-Right stride speed | -0.370 | <0.001 |
| Gait-Left stride speed | -0.405 | <0.001 |
| Gait-Left swing speed | -0.395 | <0.001 |
| Gait-Speed | -0.462 | <0.001 |
| Gait-Right swing speed | -0.385 | <0.001 |
| Gait-Left stride length | -0.400 | <0.001 |
| Gait-Right stride length | -0.367 | <0.001 |
| Gait-Recording time | 0.540 | <0.001 |
| GCD-Left stride speed | -0.368 | <0.001 |
| GCD-Left swing speed | -0.325 | <0.001 |
| Eye tracking | | |
| GPS-Completion time (fast) | 0.470 | <0.001 |
| GPS-Completion time (average) | 0.107 | 0.287 |
| AS-Correct error rate | -0.196 | 0.05 |
| OPS-Completion time (average) | 0.291 | 0.003 |
| GPS-Latency | -0.130 | 0.196 |
| OPS-Completion time (fast) | 0.163 | 0.106 |
| AS-Accuracy | -0.149 | 0.14 |
| OPS-Latency | 0.058 | 0.566 |
| AS-Completion time (average) | 0.068 | 0.504 |
| LF-Offset time accuracy (<4°) | -0.188 | 0.061 |

**Table S10** Diagnostic metrics of digital screening tools and their combined models in distinguishing patients with CI from NC (years of education ≤6)

| Diagnostic models | AUC | Accuracy | Sensitivity | Specificity |
| --- | --- | --- | --- | --- |
| *NC vs CI (cognitive impairment) without covariates* | | | | |
| Digital drawing | 0.862 | 0.735 | 0.720 | 0.750 |
| Eye tracking | 0.741 | 0.695 | 0.621 | 0.767 |
| Eye tracking& Digital drawing | 0.914 | 0.827 | 0.800 | 0.857 |
| AD8 | 0.829 | 0.815 | 0.754 | 0.877 |
| MMSE | 0.830 | 0.824 | 0.862 | 0.786 |
